# Operationalizing multimorbidity and autonomy for health services research in aging populations - the OMAHA study

**DOI:** 10.1186/1472-6963-11-47

**Published:** 2011-02-25

**Authors:** Martin Holzhausen, Judith Fuchs, Markus Busch, Andrea Ernert, Julia Six-Merker, Hildtraud Knopf, Ulfert Hapke, Beate Gaertner, Ina Kurzawe-Seitz, Roswitha Dietzel, Nadine Schödel, Justus Welke, Juliane Wiskott, Matthias Wetzstein, Peter Martus, Christa Scheidt-Nave

**Affiliations:** 1 Charité - Universitätsmedizin Berlin, Charitéplatz 1, D-10117 Berlin, Germany; 2 Robert Koch Institute, General-Pape-Str. 62-66, D-12101 Berlin, Germany; 3 Universitätsklinikum Freiburg, Sektion Tumorepidemiologie, Elsässerstr. 2, D-79110 Freiburg, Germany; 4 Gemeinsamer Bundesausschuss, Wegelystraße 8, D-10623 Berlin, Germany

## Abstract

**Background:**

As part of a Berlin-based research consortium on health in old age, the OMAHA (Operationalizing Multimorbidity and Autonomy for Health Services Research in Aging Populations) study aims to develop a conceptual framework and a set of standardized instruments and indicators for continuous monitoring of multimorbidity and associated health care needs in the population 65 years and older.

**Methods/Design:**

OMAHA is a longitudinal epidemiological study including a comprehensive assessment at baseline and at 12-month follow-up as well as brief intermediate telephone interviews at 6 and 18 months. In order to evaluate different sampling procedures and modes of data collection, the study is conducted in two different population-based samples of men and women aged 65 years and older. A geographically defined sample was recruited from an age and sex stratified random sample from the register of residents in Berlin-Mitte (Berlin OMAHA study cohort, n = 299) for assessment by face-to-face interview and examination. A larger nationwide sample (German OMAHA study cohort, n = 730) was recruited for assessment by telephone interview among participants in previous German Telephone Health Surveys. In both cohorts, we successfully applied a multi-dimensional set of instruments to assess multimorbidity, functional disability in daily life, autonomy, quality of life (QoL), health care services utilization, personal and social resources as well as socio-demographic and biographical context variables. Response rates considerably varied between the Berlin and German OMAHA study cohorts (22.8% vs. 59.7%), whereas completeness of follow-up at month 12 was comparably high in both cohorts (82.9% vs. 81.2%).

**Discussion:**

The OMAHA study offers a wide spectrum of data concerning health, functioning, social involvement, psychological well-being, and cognitive capacity in community-dwelling older people in Germany. Results from the study will add to methodological and content-specific discourses on human resources for maintaining quality of life and autonomy throughout old age, even in the face of multiple health complaints.

## Background

Multimorbidity, e. g. the concurrent existence of multiple health problems in the same person, is a highly prevalent phenomenon in old age and of growing public health impact in aging societies [1-3]. The terms multimorbidity and comorbidity have been used interchangeably. In fact, comorbidity is the older concept and was first introduced by Feinstein who demonstrated that comprehensive assessment of concomitant health problems among patients with a particular index disease is crucial to explain differences in therapeutic outcome [4].

Due to methodological challenges and limited epidemiological data, the prevalence, patterns, determinants, correlates, and consequences of multimorbidity are not well researched [5,6]. A first and major challenge lies in the definition of multi- and comorbidity itself. There is no consensus yet as to which health conditions should be considered and how exactly they should be assessed, summarized and weighted in order to arrive at some overall measure of burden of illness. Apart from quantitative aspects, the type and patterns of concurrent morbidities will matter with respect to treatment options and prognosis. A second challenge relates to measuring the impact of multi- and comorbidity [7,8]. As many of the health complaints people face in old age are chronic and progressive, and as multiple concurrent conditions are known to interact, measures of disease-specific treatment success, such as 'cure' or reduction in some surrogate measure may not be appropriate. The focus of geriatric outcome research is hence on functional measures, such as critical exhaustion of specific body functions (often termed as 'frailty'), functional disability in daily life, and social participation on one hand and on subjective measures, such as quality of life (QoL) and self-determination (autonomy) on the other [9,10]. However, there is an ongoing debate within health sciences on how to define these constructs, and on which instruments should be used to assure standardized assessment and comparability of study results [11-13]. A third and not too minor challenge lies in the fact that the relationship between multimorbidity and various outcomes is likely to be modified by a number of medical as well as non-medical resources and risk-factors. This concept has been referred to as patient complexity [14]. Examples for personal resources relevant to maintain autonomy and quality of life despite functional impairment and reduced health status include health-related knowledge, beliefs, competences, and proactive behaviour [15]. External resources include perceived social support, living conditions, and quality of health care [14]. Beyond that, context variables such as socio-demographic and cultural background as well as critical life events at personal or societal level need to be considered [16,17].

Against this background, the Federal Ministry of Education and Research (BMBF) launched a long term research initiative on 'Health in Old Age'. A total of six research consortia qualified for an initial three-year funding period (2008-2010) to provide insight into the epidemiology and socioeconomic consequences of multimorbidity in older people in Germany [18]. Among these, the Berlin-based consortium Autonomy Despite Multimorbidity in Old Age (AMA) focuses on resources and potentials to maintain everyday-functioning and self-determination of older people with multiple health constraints [19].

The population-based AMA subproject OMAHA (Operationalizing Multimorbidity and Autonomy for Health Services Research in Aging Populations) aims to develop a conceptual framework and a set of standardized instruments and indicators for continuous monitoring of multimorbidity and associated health care needs in the population 65 years and above. Main specific goals are: (1) to develop an algorithm for the comprehensive assessment of multi- and comorbidity, (2) to analyze patterns, correlates, determinants, and consequences of multi- and comorbidity; (3) to evaluate an innovative instrument for preference-based QoL assessment among elders with multimorbidity; (4) to examine the effectiveness and efficiency of different recruitment strategies and to characterize difficult-to-reach subgroups of the population 65 years and older. We describe here the design, methods, study population, and data base of the OMAHA study.

## Methods/Design

### Study design

The OMAHA study is designed as a population-based longitudinal epidemiological study of multimorbidity in the population aged 65 years and older. With regard to methods applied for continuous nationwide health monitoring in Germany [20], the OMAHA project uses two different modes of data collection and sampling frames: (1) assessment by standardized computer-assisted personal interview (CAPI) and examination in a geographically defined population sample of men and women 65 years and older residing in the inner district of Berlin (Berlin-Mitte) as of July 15, 2008; (2) assessment by standardized computer-assisted telephone interview (CATI) in a nationwide population sample of men and women aged 65 years and older who participated in previous German Health Telephone Surveys and had agreed to be re-contacted. In each case, the study protocol consisted of a comprehensive assessment at baseline and at 12-month follow-up. In addition, short intermediate telephone follow-up interviews for assessment of vital and functional status were conducted at month 6 and 18.

The study was approved by the local ethics committee at Charité - Universitätsmedizin Berlin and conducted in compliance with data protection and privacy regulations as requested by the Federal and Berlin Offices for the Protection of Data. Study participants were informed in detail about the study objectives, interview and examination procedures as well as pseudonymized record keeping and subsequent data analysis. Persons participating in the personal interview and examination gave written informed consent prior to study inclusion. A subset also provided written permission to contact their family doctor for validation of specific self-reported medical conditions (e. g., ischemic heart disease, diabetes, asthma, chronic bronchitis). Oral informed consent to study participation was obtained from participants in previous German health telephone surveys before conducting the OMAHA baseline CATI.

### Sampling procedures and recruitment of study participants

For the Berlin OMAHA cohort, we drew an age (65-69, 70-74, 75-79, 80-84, 85+ years) and sex stratified random sample of 2000 men and women aged 65 years and older from the official register of residents in Berlin-Mitte as of July 15, 2008. All individuals of the population sample who were then verified as alive, still living in Berlin-Mitte and available during the study recruitment period were eligible for the study. Exclusion criteria were: death, permanent change of residence outside of Berlin-Mitte or to an unknown address, and continuous absence from Berlin during study recruitment period. Anticipating an overall response rate of about 20% and an overall proportion of unverifiable contacts of 5-10%, the total sample size was sufficiently large to achieve a study population of n = 300 for the main study and of n = 100 for preceding pre-test evaluation.

To assure public support of the study, we communicated the study goals and logistics to members of the Berlin Medical Association via the official journal of the Berlin Medical Association (Anonymous, 2009) and to community officials (e. g., local police stations, churches) via telephone contacts.

Individuals belonging to the main sample were initially contacted by post. The letters contained a brief description of the study, an invitation to participate, and a prepaid self-addressed envelope together with a return sheet to fill in telephone numbers, preferred contact times, and choice of home or study centre visit for the assessment. Participants were offered a small monetary incentive (€ 10) plus reimbursement for travel expenses. Foreign nationals were offered a brief description of the study in seven different languages (Turkish, Russian, Arabic, Serbian, Croatian, Polish, and English).

In order to identify and further characterize persons who did not participate, we explicitly asked family members or caregivers to respond in case that an eligible person was not able to participate due to cognitive impairment or severe illness and to consent to proxy telephone interviews.

Among persons who did not respond to the initial mailed invitation, we conducted a randomized controlled trial which evaluated the effectiveness and efficiency of three different intensified recruitment strategies: (a) personal visits, (b) telephone calls, or (c) mailed reminder letters including the initial invitation letter and enclosures. Statistical analysis of this comparative evaluation of recruitment strategies is completed and results are currently prepared for publication in a separate paper.

The *German OMAHA cohort *was recruited from a pool of persons who had previously participated in the German Telephone Health Surveys 2004 or 2006. Nationwide telephone health surveys have been conducted in Germany annually by the Robert Koch Institute since 2002/2003; sampling procedures and recruitment strategies have been previously described in detail [21]. In brief, telephone numbers from complete listings of landline telephone connections belonging to private households in Germany are randomly generated, applying the Gabler-Häder method [22]. This method assures that households with unregistered telephone numbers are included in the 'target sample' of telephone surveys. Random sampling at the individual level is achieved by the 'next-birthday-method', i.e. only the adult whose birthday is coming up next to the date of first contact to the respective household is included in the target sample. Automated redialling systems and computer-assisted interviewer guidance assure that telephone contact and interview procedures are conducted in a highly standardized and efficient way. In the 2004 and 2006 German Telephone Health Surveys, 56.1% and 56.6% of contacted persons 18 years of age and older completed the survey (3376 men; 3965 women in 2004 [23]; 2682 women and 2860 men in 2006 [24]). The majority of survey participants 65 years and older (n = 1263 or 87.4% in 2004, and n = 846 or 86.0% in 2006) consented to be re-contacted by the Robert Koch Institute for future health surveys. Excluding n = 557 persons who were part of a random sample included in a European telephone health survey, a total of n = 1552 men and women remained to be re-contacted for the OMAHA baseline telephone assessment.

### Response and analysis of non-response bias

**Figure 1:**
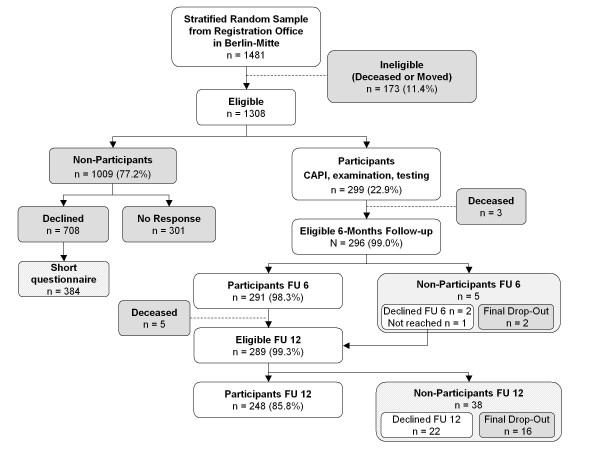
**Response in the Berlin OMAHA cohort**.

Of the total Berlin sample, 519 persons (26.0%) had initially been contacted for preceding pre-test evaluation, leaving a total of n = 1481 persons for the main study (Figure 1). Of these, 173 persons (11.7%) were excluded as they had died (n = 68), had changed residence (n = 85), or were absent during the entire recruitment period (n = 20). Of the 1308 eligible persons, 299 (22.9%) participated in the study and completed the full baseline assessment between January and June 2009. Among 1009 non-participants, n = 384 (38.1% of non-participants) declined full assessment but completed a short questionnaire (also available in the seven different languages mentioned above) covering self-rated health, limiting longstanding illness, disability, care dependency, living arrangements and marital status; n = 324 (32.1% of non-participants) declined both full assessment and short questionnaire; and n = 301 (29.8%) did not respond to all our contacts. The short questionnaires were administered via standardized telephone interview or self-administered postal questionnaires which were returned in a self-addressed prepaid envelope. Among n = 296 participants surviving to the 6-month-follow-up, n = 291 (98.3%) completed the brief 6-month follow-up questionnaire delivered by standardized telephone interview. The 12-month follow-up assessment was conducted between January and April 2010 and is complete for n = 248 (85.2% of 291 participants surviving to 12 months). The concluding 18-month telephone follow-up was completed in October 2010 for n = 262 participants (91.0% of 288 participants surviving to month 18).

For preceding pre-test evaluation, a random sample of n = 100 stratified by sex and five age groups (65-69, 70-74, 75-79, 80-84, 85+ years) was drawn from the German Telephone Health Survey sample 2007 [25].

**Figure 2:**
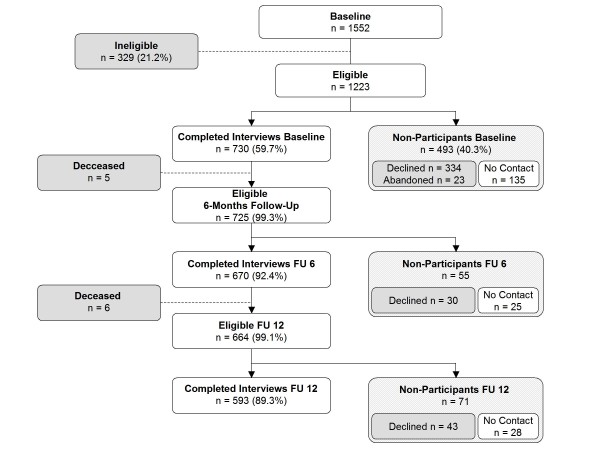
**Response in the German OMAHA cohort**.

N = 1552 participants in previous German Telephone Health Surveys 2004 and 2006 were selected for the OMAHA study (Figure 2), 329 persons (21.2%) were excluded because they had died or their telephone numbers were found to be disconnected. Of the remaining 1223 persons, 730 (59.7%) completed the OMAHA baseline assessment via computer-assisted telephone interview (CATI) between March and April 2009. Among non-participants, the majority (n = 334) refused to participate, while some persons (n = 23) started but did not complete the telephone interview, and n = 135 (27.5%) could not be reached during the baseline study period. Of baseline participants, n = 670 (91.8%) completed the brief 6-month telephone follow-up, administered via CATI. Among the 60 persons not continuing into follow-up, 5 had died in the interim, 30 refused to participate (11 stating that decision directly after baseline assessment), and 25 could no longer be reached by the given telephone number. The full 12-month follow-up assessment was conducted in May and June 2010 and was completed for n = 593 (81.2%) of OMAHA baseline study participants (Figure 2). The concluding 18-month telephone follow-up is currently underway and will be completed by January 2011.

Non-response analyses will be conducted based on comparisons of key health and socio-demographic characteristics between participants and non-participants. For the nationwide cohort such information can be extracted from data collected during previous German Telephone Health Survey contacts [23,24]. In the Berlin cohort, information on age, sex, nationality, nursing home residence, and area deprivation score was available from the dataset provided by the residents registration office in Berlin-Mitte and official social statistics for the city of Berlin. Further comparisons between participants and non-participants with respect to health and disability as well as social networks and care dependence will be limited to the 38% subset of non-participants who answered a brief questionnaire for baseline assessment.

### Constructs and instruments

**Figure 3:**
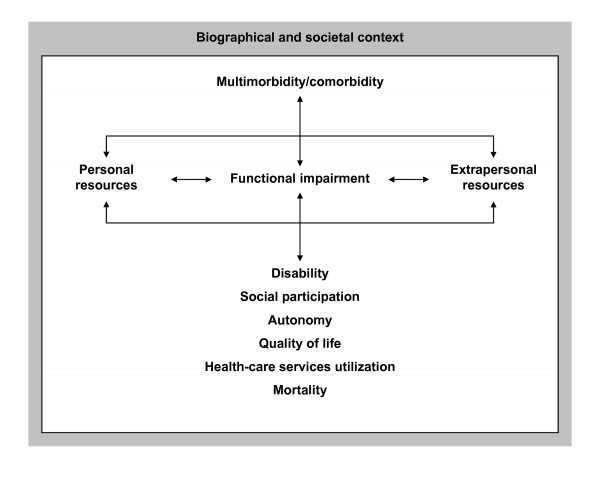
**Framework of the OMAHA Study**.

Within the conceptual framework depicted in Figure 3, we selected and composed a set of instruments that would permit to provide valid, reliable, and efficient measures of (a) *multimorbidity*, (b) potential consequences of multimorbidity, including *impairment of body functions and frailty*, *autonomy*, *quality of life*, and *health care services utilization*, (c) personal and social *resources *likely to modify the association between multimorbidity and outcome measures, (d) socio-demographic and biographical context variables.

Selection of instruments was based on extensive literature review and feasibility pre-testing. Table 1 provides an overview of the main theoretical constructs, construct domains, and instruments. Assessment tools were aligned as closely as possible between the two OMAHA study arms (Berlin cohort and German cohort). Nevertheless, constructs covered and instruments used for their measurement differed to some degree according to the different assessment modes.

**Table 1:** Constructs, facets, and measurement tools in OMAHA

Theoretical Construct and Facets	Measure	Berlin cohort	German cohort
**Multimorbidity**			
Long-standing or chronic disease	Minimum European Health Module (MEHM)[31,32]	PI	TI
Diseases and health problems	Closed-ended questions on medical conditions and health problems (cf. Figure 2) ^a^	PI	TI
Operations	Open-ended questions for surgery since age 40, followed by closed-ended questions ^a^	PI	TI
Regular medication	Closed-ended question ^a^	PI	TI
Depression	Patient Health Questionnaire (PHQ2/9)[33,34]	PI	TI
Medication	Barcode scanning and assessment of currently used medication[20]	PI	-
Falls	Closed-ended questions according to[35]	PI	TI
Fractures	List ^a^	PI	-
Medical care	According to German National Health Interview and Examination Survey[30]	SQ	TI
Blood pressure	Blood pressure meter ^b^	PI	-
Weight	Electronic scales ^c^	PI	-
Height	Portable stadiometer ^d^	PI	-
Circumference of waist, calf, arm	Flexible measuring tape ^e^	PI	-

**Quality of Life**			
Self-rated overall health	Minimum European Health Module (MEHM; also question 2 in SF-36)[31,32]	PI	TI
Global life-satisfaction	Single question ^a^	PI	TI
Global life-satisfaction	Satisfaction With Life Scale (SWLS)[36]	PI	-
Global life-satisfaction	Fragebogen zur Lebensqualität multimorbider älterer Menschen (FLQM)[37]	PI	-
Subjective well-being	International Positive and Negative Affect Schedule (IPANAS)[38,39]	PI	-
Comparative self-rated overall health	Closed-ended questions ^a^	SQ	-
Health-related quality of life	EQ-5D[40,41]	SQ	TI
Subjective well-being	Vitality sub-scale of SF-36[42,43]	SQ	TI
Pain	Questions 7 and 8 of SF-36; adapted time-frame[42,43]	SQ	TI

**Autonomy**			
Limitations due to health problem	Minimum European Health Module (MEHM)[31,32]	PI	TI
Need for assistance	Closed-ended questions modified from[44]	PI	TI
Functional health I: ADL	According to Katz[45,46]	PI	TI
Functional health I: IADL	According to Lawton & Brody[47,48]	PI	TI
Functional health II: Functional limitations	Closed-ended questions adapted from[32]	SQ	TI
Autonomy	Wahrgenommene Autonomie im Alter (WAA)[49]	SQ	-

**Resources (incl. Socio-Demography) and Performance Tests**			
Year of birth	According to German National Health Interview and Examination Survey[30]	PI	TI
Gender	According to German National Health Interview and Examination Survey[30]	PI	TI
Marital status	According to German National Health Interview and Examination Survey[30]	PI	TI
Education and employment	According to German National Health Interview and Examination Survey[30]	PI	TI
Net household income	According to German National Health Interview and Examination Survey[30]	PI	TI
Household size	Closed-ended questions [24]	PI	TI
Number of and contact with children	Closed-ended questions[44]	PI	TI
Religion	Closed-ended question [50]	PI	TI
Migration	According to Schenk et al.[51]	PI	TI
Health insurance	According to German National Health Interview and Examination Survey[30]	SQ	-
Nursing care level	Closed-ended question ^a^	PI	TI
Housing and environment	Mannheimer Inventar der Lebensverhältnisse im Alter (MILVA), subscale, housing'[52]	SQ	TI
Social contacts	Mannheimer Inventar der Lebensverhältnisse im Alter (MILVA), subscale, contacts'[52]	SQ	TI
Social support	Berliner Social Support Scales (BSSS)[53]	SQ	-
Nutrition	Mini Nutritional Assessment (MNA)[54]	PI	TI
Changes in height and weight	Open-ended questions ^a^	PI	TI
Healthcare products	List ^a^	PI	TI
Alcohol consumption	Alcohol Use Disorders Identification Test - alcohol consumption questions (AUDIT-C)[55,56]	SQ	TI
Smoking	According to German National Health Interview and Examination Survey[30]	SQ	TI
Medication addiction	Severity of Dependence Scale (SDS)[57,58]	SQ	-
Physical activity	Closed-ended questions ^a^	SQ	TI
Activities	List ^a^	SQ	-
Critical life-events	List following[59]	SQ	-
Coping	Proactive Coping Inventory PCI[15]	SQ	-
Cognitive function: Episodic memory	Memory Impairment Screen (MIS)[26,60,61]	PI	TI
Cognitive function: Verbal fluency	Verbal fluency subtest of the CERAD test battery[62,63]	PI	TI
Cognitive function: psychomotor speed	Digit symbol substitution test of WAIS (adapted German version from BASE)[64,65]	PI	-
Cognitive function: Temporal orientation	Subscale orientation of the ADAScog[26,66]	PI	TI
Grip Strength	Smedley dynamometer[67,68]	PI	-
Functional Mobility	Timed Up and Go-Test[27]	PI	-
Lower extremity function	Chair-Rise-Test[28]	PI	-
Balance	According to Guralnik[29]	PI	-

PI = personal interview/assessment; SQ = self-complete questionnaire; TI = telephone interview

^a ^Self-developed questionnaire.

^b ^Datascope Accutor Plus, the Netherlands.

^c ^SECA, Germany, accuracy of measurement 0.1 kg.

^d ^Holtain Ltd., UK, accuracy of measurement 0.1 cm.

^e ^Hoechstmass, Sulzbach, Germany, accuracy of measurement 0.1 cm.

**Figure 4:**
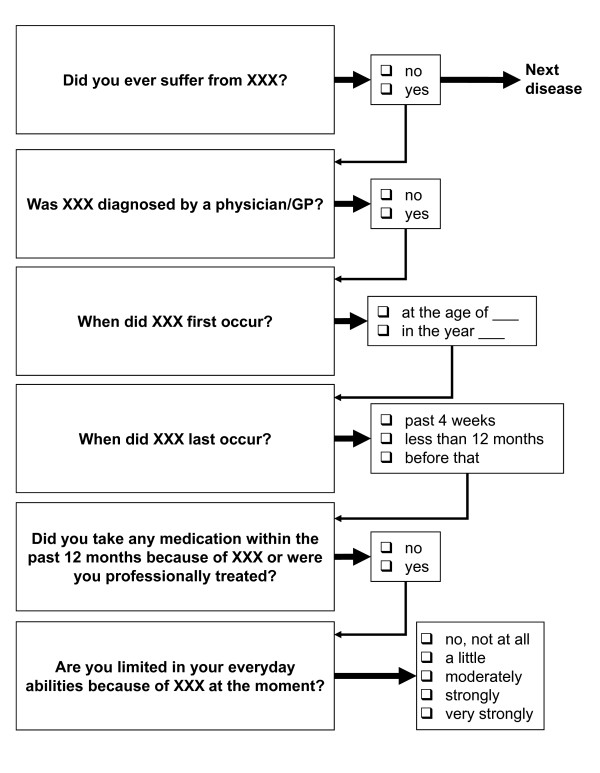
**Assessment of morbidity in OMAHA**.

The theoretical construct of *multimorbidity *is composed of two domains: (a) health status as assessed by self-reported physician-diagnosed health conditions, history of surgical procedures, history of fractures past age 50 years, medication use within 7 days prior to the interview, and standardized measures (Berlin cohort only) of height, weight, waist and calf circumference, and blood pressure; (b) self-reported current symptoms and complaints including sensory limitations (vision and hearing problems), urinary and faecal incontinence, constipation, back pain, and joint complaints. Overall, 31 specific health conditions were covered in the CAPI and CATI following a standardized sequence of questions on first and last time of occurrence of symptoms, medical treatment within the past 12 months, and perceived level of condition specific limitation in daily life (Figure 4). Current symptoms of depression as assessed by the Patient Health Questionnaire (PHQ) were used as an indicator of mental health. Operations, falls (only Berlin cohort), and health services utilization were also assessed. Functional assessment covered (a) a short neuropsychological test battery, (including tests of orientation, episodic memory, verbal fluency, and psychomotor speed) and (b) standardized measures of physical functioning (including measures of grip strength, balance, and lower extremity dysfunction. The digit symbol substitution test (DSST) and physical functioning tests were restricted to the Berlin OMAHA study cohort. Conceptualizing frailty as a critical exhaustion of specific body functions or combinations of body functions we used previously published cut-off levels [26-29]. In this context, self-reported weight loss within the past 12 months, height loss since age 25 years, and history of falls within the past 12 months and the past four weeks were also assessed.

Standardized anthropometric measurements and automated assessment of current medication use were limited to the Berlin OMAHA study cohort. A complete inventory of all medications used within 7 days prior to the interview (including prescriptions as well as non-prescription drugs) was assured by scanning the original containers brought to the examination site for that purpose. All drugs were automatically coded by an underlying software system to the WHO-ATC system. Details on medication use also included self-reported indications, form of administration, frequency of intake, origin of the medicine, and duration of use.

*Autonomy *is conceptualized twofold as (a) absence of disability in daily life and dependence on care, and (b) perceived self-determination. Disability and dependence on care were assessed by standardized CAPI or CATI, covering questions on limitations in activities of daily living (ADL, IADL), and everyday competence. Questions on subjective self-determination were covered in the self-administered questionnaire and are hence limited to the Berlin cohort.

*Quality of life *comprises the following domains: self-rated health, subjective well-being and vitality, pain, health-related QoL, global life satisfaction, and preference-based quality of life. All of these except preference-based quality of life were assessed in both OMAHA cohorts by validated instruments via standardized interview (CAPI or CATI) or self-administered questionnaire. Preference-based QoL was assessed only in the Berlin cohort by the newly developed tool FLQM ('Fragebogen zur Lebensqualität multimorbider älterer Menschen') as part of the CAPI. Additional instruments to measure global life satisfaction (Satisfaction with Life Scale, SWLS) and subjective well-being (International Positive and Negative Affect Schedule, IPANAS) were administered in the Berlin cohort only.

*Resources *can roughly be divided into personal, social and organizational resources. As facets of personal resources, individual health-related behaviours (alcohol consumption, nutrition, tobacco use, physical activity), educational status, and income as well as housing and environment were assessed in both OMAHA cohorts by standardized self-administered questionnaire or CATI. Proactive coping as another important personal resource was assessed by the Proactive Coping Inventory (PCI) via self-administered questionnaire in the Berlin cohort only, since the PCI is not suitable for administration via CATI. As part of the social resource domain, social contacts were assessed in both OMAHA cohorts by the same validated instrument (MILVA). In lack of a validated instrument for administration by CATI, the assessment of subjective social support (by BSSS) was restricted to the Berlin OMAHA cohort. Organizational resources assessed were health insurance, family doctor, housing and environment, and nursing care level within the German healthcare system. Additional context variables covered age, critical life events, and migration background.

### Power analysis

Due to the exploratory nature of the study and the multiplicity of outcomes, no power calculation was considered.

### Data management and preparation for analysis

A statistician supervised, validated, and where necessary corrected the data throughout the process of data-collection. Cleaned final data sets were provided by the statistician within less than 6 months after the completion of data collection. A detailed documentation of the process was continuously accessible to the research team.

### Quality assurance

To achieve a high degree of standardization in the survey, the study nurses for personal interviews (CAPI) were initially trained and continuously supervised. Additional training sessions took place before the 12-months follow-up. Telephone interviewers were highly experienced in carrying out telephone health surveys and received specific training before the baseline interview as well as before the 6-month and 12-month follow-up interviews. Standard operation procedures (SOPs) were supplied for all parts of the computer-assisted interviews (CAPI and CATI), functional assessments, and anthropometric measurements. Quality standards and requirements for internal quality control were developed according to recognized epidemiological guidelines and standards applied in German Health Interview and Examination Surveys [30].

## Discussion

The OMAHA project is a longitudinal epidemiological study in two population-based cohorts of older people aged 65 years or older using different methods of data collection. In the urban cohort (Berlin cohort), an age- and sex-stratified sample of residents of an inner-city district of Berlin was assessed by face-to-face interviews, self-administered questionnaires and measurement of physical functions and body measures. In the nationwide cohort (German cohort), participants of former German Health Telephone Surveys were assessed by telephone interview.

Our research is focused on the development of a comprehensive assessment of multi- and comorbidity and the analysis of patterns, correlates, determinants, and consequences of these. Also, an innovative instrument for preference-based QoL assessment among elders suffering from multimorbidity will be evaluated. On the methodological level, we examine the effectiveness and efficiency of different recruitment strategies and will characterize difficult-to-reach subgroups of the population 65 years and older. Data collection comprises four waves in each of the two cohorts: two more extensive waves at baseline and 12-month follow-up, and two very brief waves at months 6 and 18 from baseline assessment. This opens up a wide range of opportunities for analyses on trajectories of health states, longitudinal relationships of outcome determinants, and, most importantly, causal relationships between conditions and trajectories over a period of 18 months.

The parallel application of instruments in the two cohorts and modes of administration (CAPI and CATI) will on the one hand allow us to identify those tools that are most suitable for telephone administration. On the other hand, we will also be able to make comparisons between the urban Berlin cohort and the nationwide cohort with respect to selected variables cross-sectionally as well as longitudinally. The wide variety of measures relating to similar constructs allows us to soundly determine the psychometric properties of adaptations and new developments for geriatric and gerontological assessments in older age groups. After the completion of analyses relating to validity and reliability of (1) adaptations of existing measurement tools and (2) the newly developed quality of life assessment tool (FLQM) we will be able to provide a wide-ranged set of instruments and tools for personal and telephone assessment in population-based health surveys.

Clearly, the samples are subject to selective inclusion. Within the Berlin cohort, there were considerable differences in sample structure with respect to different major sub-populations, such as migrants or people living in nursing-homes and socio-economically deprived areas. In addition to the specified sub-populations, people suffering from dementia or cognitive impairment and those who are heavily restricted in their sensory capacity are not likely to be adequately represented in both cohorts. Because determining barriers and evaluating recruitment strategies are among our goals, we did not expect the participating sample to be representative. In contrast, analyses of the effectiveness and efficiency of the three different recruitment strategies in the Berlin cohort and detailed analysis of participant/non-participant characteristics in both cohorts will enable us to derive strategies on how to get better access to, and response from, these groups. In future studies, a strategy of oversampling underrepresented, yet notably prevalent and politically important groups could serve as one means to minimize selection bias.

OMAHA offers a wide spectrum of data related to health, functioning, social involvement, psychological well-being, and cognitive capacity throughout old age in Germany, opening up new opportunities for further gerontological and geriatric research. Results from the study will add to methodological as well as content-specific discourses on human resources for maintaining high levels of autonomy and quality of life in old age, even in the face of multiple health burdens and morbidity.

## Competing interests

The authors declare that they have no competing interests.

## Authors' contributions

MH and CSN conceptualized and supervised the study, analyzed the data and wrote the final manuscript. JF, MB and PM conceptualized and supervised the study, analyzed the data and made substantial contributions to the writing of the final manuscript. JWelke, JSM, HK, UH assisted in the conceptualization of the study, provided specific knowledge, made substantial contributions to the acquisition and quality assurance of the data and contributed to the final manuscript. RD, NS, IKS, JWiskott provided specific knowledge, made substantial contributions to the acquisition and quality assurance of the data and contributed to the final manuscript. AE assisted in the conceptualization of the study, analyzed the data and contributed to the final manuscript. BG analyzed the data and contributed to the final manuscript. MW made substantial contributions to the acquisition and quality assurance of the data and contributed to the final manuscript.

## Pre-publication history

The pre-publication history for this paper can be accessed here:

http://www.biomedcentral.com/1472-6963/11/47/prepub
